# Analysis of complete mitochondrial genome sequence of *Pontania dolichura* (Hymenoptera: Tenthredinidae) (Thomson, 1871)

**DOI:** 10.1080/23802359.2022.2078237

**Published:** 2022-06-07

**Authors:** Guo Sun, Chengbo Liang, Jingyan Yan, Yue Zhang, Yahuan Shui, Yuantao Zhou

**Affiliations:** College of Agriculture and Animal Husbandry, Qinghai University, Xining, China

**Keywords:** *Pontania bridgmannii*, mitochondrial genome, sequence, phylogenetic analysis

## Abstract

*Pontania dolichura* is a leaf-eating pest that mainly damages willow trees and is widely distributed in northern regions. In this study, we sequenced the entire mitochondrial genome of *P. dolichura* (GenBank accession number: MZ726800). The circular gene was 16,104 bp in length and comprised 38 column elements, including 13 protein-coding genes (PCGs), 22 transfer RNA genes, two ribosomal RNA genes, and a non-coding control region. Most of the PCGs of *P. dolichura* have typical ATN (Met) start codons and typical TAN stop codons. The A + T contents of the genome, PCGs, transfer RNAs (tRNAs), and ribosomal RNAs (rRNAs) were 80.32%, 78.66%, 81.94%, and 82.59%, respectively. Phylogenetic analysis supported the close genetic relationship between *P. dolichura* and *Mesoneura rufonota* indicating that the two species share more recent common ancestor gene. These data will be useful for further molecular identification and population genetics studies.

*Pontania dolichura* belongs to the Tenthredinidae family (subfamily: Nematinae; order: Hymenoptera). It is harmful to willow trees, especially weeping willows, which are widespread in north, northwest, and northeast China (Liu 2011). It feeds on the leaves, causing the affected parts to gradually swell and form insect galls, thereby hindering the growth and development of the tree and affecting the landscape greening effect and ecological function (Qi et al. 2020). The current research on sawflies includes the spatial pattern of the larvae of *P. dolichura* to determine the control methods and forecasting investigations according to regular morphological change patterns to determine the best control period and other aspects (Zhang et al. 2006; Xue et al. 2017). In this study, we sequenced the entire mitochondrial genome of *P. dolichura* to provide information for the discovery of better markers in this family classification and as a useful reference to prevent damage to trees (Figure 1).

**Figure 1.**
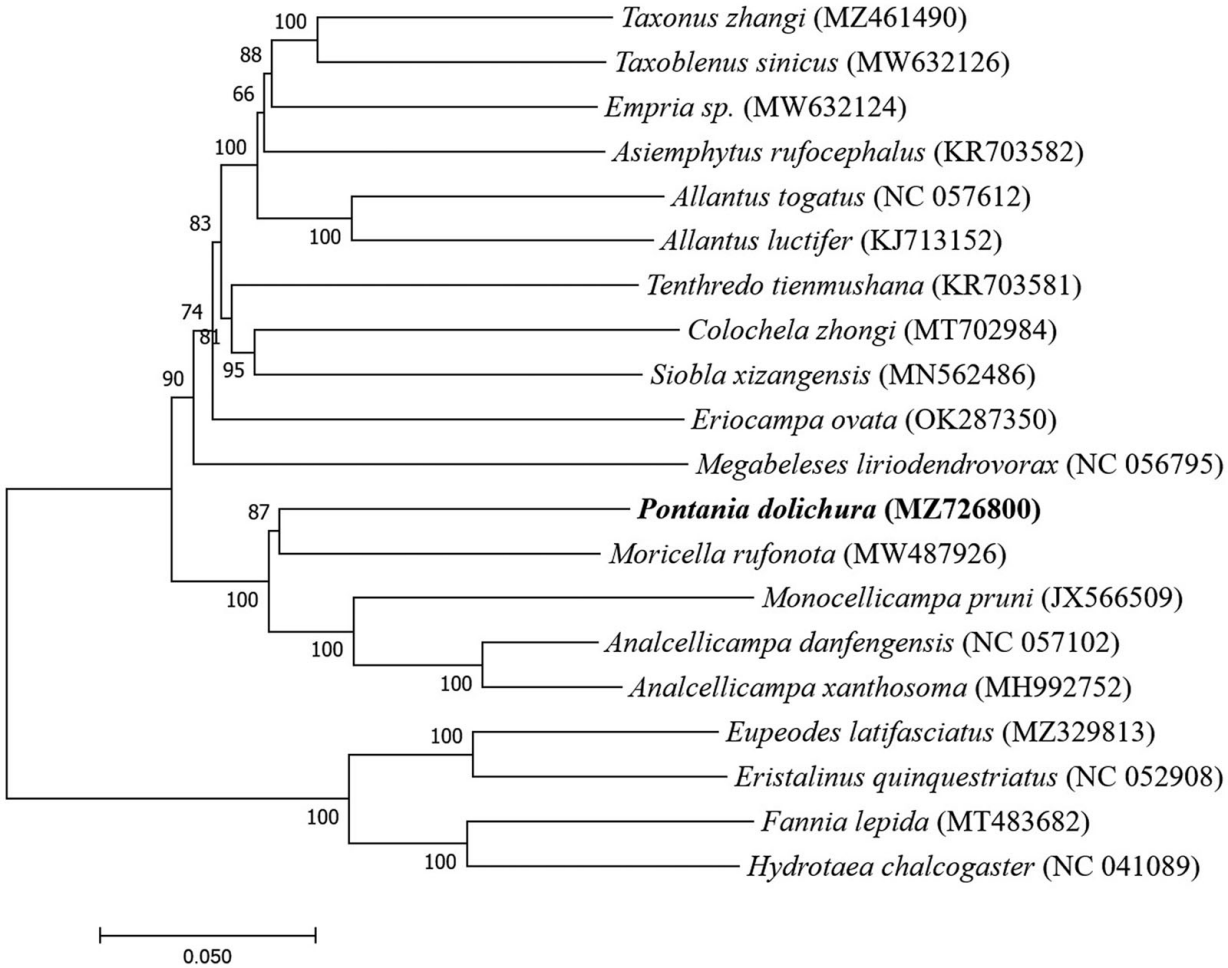
The Neighbor-joining phylogenetic tree based on 20 mitochondrial genome sequences.

Samples of *P. dolichura* adults were obtained from Xining, Qinghai Province, China (36°62N, 101°77E) in May 2021 and stored in the Insect Collection of the Entomology Lab, College of Agriculture and Animal Husbandry, Qinghai University with specimen accession number ZYT-202105-02 (email: 1282186715@qq.com). Mitochondrial genomic DNA (mtDNA) was extracted from a single sample. The NovaSeq Sequencing System (Illumina, San Diego, CA) was used to determine the complete mtDNA sequence with a read length of 150 bp. SPAdes v3.10.1 software (http://cab.spbu.ru/software/spades/) (Bankevich et al. 2012) was employed to assemble the mitogenome. Gaps were filled with SSPACE 3.0 (Boetzer et al. 2011) and GapFiller 1.1 (Boetzer and Pirovano 2012). The assembled sequences were annotated using the MITOS web server (Bernt et al. 2013).

The mitochondrial genome (GenBank accession number: MZ726800) of *P. dolichura* is 16,104 bp long and has a typical structure. The genome contains 38 sequence elements, including 13 protein-coding genes (PCGs), 22 transfer RNA (tRNA) genes, two ribosomal RNA (rRNA) genes, and a non-coding control region (D-loop region). This profile is similar to the composition of the mitochondrial genome of classic insects. A, T, G, and C contents of the genome were 43.14%, 37.18%, 7.67%, and 12.02%, respectively. The ratio of A to T (80.32%) was higher than that of G and C (19.68%).

The PCGs of *P. dolichura* display typical ATN (Met) start codons for invertebrate mitochondrial PCGs. They comprise two ATA genes (cox1 and nad4), six ATT genes (nad1, nad2, nad3, nad5, nad6, and atp8), and five ATG genes (cox2, cox3 atp6, cob, and nad4). Twelve PCG genes terminate with the typical TAN stop codons that have a TAA stop codon, three genes (atp8, nad3, and cob) have a TAG stop codon, and one gene (nad4) ends with the incomplete codon T, which may be formed after transcription through the action of poly adenylate to complete transcription termination (Ojala et al. 1981). The determined A + T contents of the *P. dolichura* genome, PCGs, tRNAs, and rRNAs were 80.32%, 78.66%, 81.94%, and 82.59%, respectively.

Based on sequencing results of the mitochondrial genome and the genome sequence obtained from GenBank, we studied the phylogenetic relationship between *P. dolichura* and the other 19 species. Alignment was performed using MAFFT7. A neighbor-joining tree was constructed using MEGA7 (with 1000 bootstrap repeats) (Katoh and Standley 2013; Kumar et al. 2016). Sixteen species belonged to Hymenoptera, while four were outgroups (Diptera: Muscidae and Syrphidae). The phylogenetic tree showed that all 15 Hymenoptera species were first clustered into the same large branch. We found that *P. dolichura* easily formed a clear orthologous lineage with the *M. rufonota*, with a bootstrap rate of 87%, indicating that they are more closely related and the two species share more recent common ancestor gene (Figure 1). We also found that the four species of insects with Diptera as the outgroups were clustered into another subclade alone. Our study describes the mitogenome of *P. dolichura* and reconstructs the phylogenetic relationship. These data will be useful for further molecular identification and population genetic studies of this species.

## Ethical approval

The study does not involve humans or animals. This study does not need ethical approval or permissions to collect the sample, which is a pest and we need proper control.

## Author contributions

Guo Sun, Chengbo Liang, Jingyan Yan, and Yuantao Zhou were involved in the conception and design, analysis and interpretation of the data; Yue Zhang and Yahuan Shui collected insects; Guo Sun drafted the paper; Yuantao Zhou revised it critically for intellectual content. Yuantao Zhou and Guo Sun gave final approval of the version to be published. All authors agree to be accountable for all aspects of the work.

## Data Availability

The genome sequence data that support the findings of this study are openly available in GenBank of NCBI at https://www.ncbi.nlm.nih.gov/ under the accession no. MZ726800. The associated BioProject, SRA, and Bio-Sample numbers are PRJNA757542, SRR15883156, and SAMN20966672, respectively.
